# Multimodal Imaging of a Case of Monitoring of Acute Posterior Multifocal Placoid Pigment Epitheliopathy (APMPPE): Long-Term Follow-Up

**DOI:** 10.1155/crop/9924678

**Published:** 2025-02-14

**Authors:** Monika Sarna, Michal Wilczynski, Arleta Waszczykowska

**Affiliations:** Department of Ophthalmology, Medical University of Lodz, Lodz, Poland

**Keywords:** APMPPE, HLA, OCT, OCTA, treatment, vascular changes

## Abstract

An 18-year-old male presented with bilateral vision loss, worsening over 10 days in the left eye and rapidly in the right eye following a suspected viral respiratory infection. On admission, his best corrected visual acuity (BCVA) was 0.9 in the right eye and 0.025 in the left. No inflammation was found in the anterior segment or vitreous body, but both eyes showed multiple yellow–white, plaque-like lesions in the retina and choroid, with foveal involvement in the left eye. Diagnostic tests revealed choriocapillaris flow deficits (optical coherence tomography angiography (OCTA)), hyperreflective changes in the outer retina and choroidal thickening (optical coherence tomography (OCT)), hypofluorescence and patchy hyperfluorescence (fluorescein angiography (FA)), hypoautofluorescence with peripheral hyperautofluorescence (fundus autofluorescence (FAF)), reduced a- and b-wave amplitudes (electroretinogram (ERG)), and scotomas with decreased retinal sensitivity (visual field (VF)). The presence of HLA-B15 and HLA-B35 antigens was confirmed. Treatment with oral methylprednisolone and intravenous acyclovir led to significant improvement within 1 day. BCVA improved to 0.9 in the right eye and 0.25 in the left, with further improvement to 0.9 and 0.5 9 days after discharge. Full visual recovery was achieved within 5 weeks. This case underscores the diagnostic value of OCTA and suggests a potential genetic predisposition linked to HLA-B15 and HLA-B35. It also highlights the effectiveness of methylprednisolone and acyclovir in APMPPE following a viral infection.

## 1. Introduction

Acute posterior multifocal placoid pigment epitheliopathy (APMPPE) is a rare disease, first described by Gass [1] in 1968. The disease presents with sudden, bilateral vision loss, without gender predilection, more commonly affecting young and middle-aged individuals. In one-third of cases, it is preceded by a flu-like infection [2, 3]. In the pathophysiology of APMPPE, an immune system response to an infectious agent or direct cell infection through the angiotensin-converting enzyme 2 receptor is considered [3]. Cases of postvaccination occurrence of APMPPE have been described in the literature [4].

Defining the primary site of inflammation is clinically challenging due to the interdependence of the choroid and the retina. The pathophysiology of the disease is not fully understood. It is believed that inflammation leads to choriocapillaris occlusion and secondary hypoxia of the adjacent retina. The choriocapillaris nourishes the outer layers of the retina, while the retinal pigment epithelium (RPE) provides nutrients and growth factors to the retina and removes metabolites. Choriocapillaris hypoperfusion results in damage to the RPE and secondary atrophy of the choroid and adjacent photoreceptors [5, 6].

The gold standard for diagnosing APMPPE remains optical coherence tomography angiography (OCTA), which allows noninvasive assessment of blood flow through the retinal and choroidal vessels [7, 8]. Based on the evolution of choriocapillaris changes observed in OCTA, we observed the chorioretinal and transitional phases.

Treatment of APMPPE remains controversial, as many clinicians believe that pharmacotherapy should not be used due to the self-limiting nature of the symptoms [9]. The literature also contains opinions that treatment should be tailored to the patient's needs. In cases involving the macula or central nervous system, systemic steroid therapy is recommended [6, 8, 10].

## 2. Case Presentation

An 18-year-old male patient came to the Clinical Department of Ophthalmology at Norbert Barlicki University Teaching Hospital No. 1 in Lodz, Poland, due to significant bilateral loss of visual acuity. Vision impairment in the left eye had lasted for approximately 10 days and had intensified over time, while there had been a sudden deterioration of visual acuity in the right eye for 2 days. The patient reported a gray “spot” in the center of the visual field (VF) of the left eye and several smaller gray dots in the VF of the right eye. The ophthalmological symptoms were preceded by a respiratory infection of probable viral etiology. On the day of admission, the patient reported significant body weakness and headache and mentioned that he had an upper respiratory tract infection 10 days before. The patient denied having chronic diseases, taking chronic medications, contact with animals, eating raw meat, having risky sexual contacts, contact with a person suffering from tuberculosis, joint pain, or disturbing skin lesions, as well as autoimmune diseases and cancer in the family.

A week before coming to our department, the patient had an ophthalmological examination at another hospital. He reported decreased visual acuity in the left eye that had been present for several days. Slit lamp examination and ultrasound examination did not reveal any abnormalities. Best corrected visual acuity (BCVA) was 1.0 in both eyes. OCTA examination was not performed at that time. The patient received treatment with oral ascorbic acid and rutoside and pranoprofen and dexamethasone drops topically to the left eye. Clinical symptoms suggest that the patient may have been in the choroidal phase of the disease at the time.

### 2.1. Admission Day

**Figure 1:**
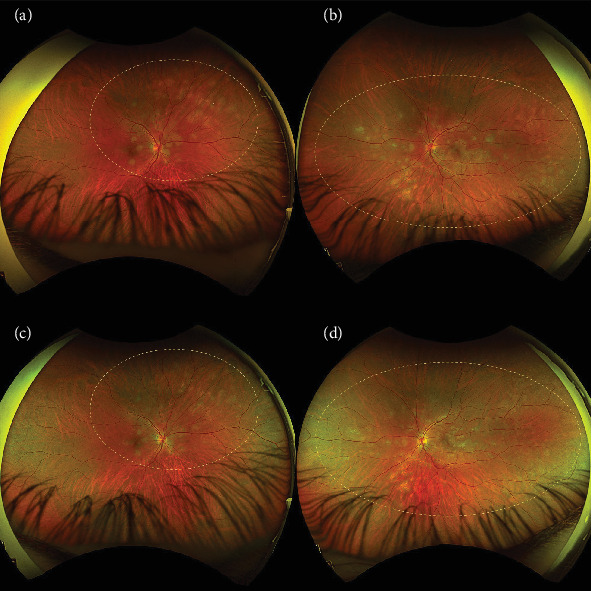
Bilateral color fundus images using Optos California showing (a) fundus of the right eye during hospitalization, showing scattered yellow–white lesions around the optic disc and in the upper hemisphere of the retina, with three prominent lesions in the macula (inside the circle). (b) Fundus of the left eye during hospitalization, showing more numerous, plaque-like lesions forming a diffuse area in the macula (inside the circle). (c) Fundus of the right eye at 5-week follow-up, showing smaller patchy lesions compared to the day of discharge (inside the circle). (d) Fundus of the left eye at 5-week follow-up, showing smaller patchy lesions compared to the day of discharge (inside the circle).

**Figure 2:**
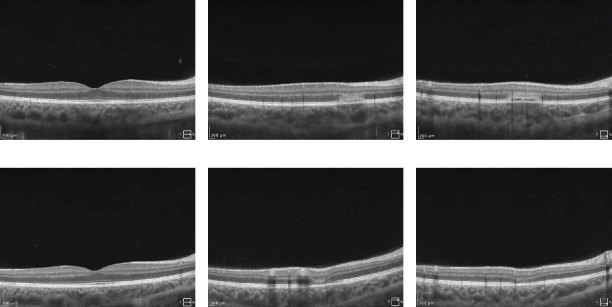
The choroid of the right eye has been cropped in the OCT images. Images showing active, hyperreflective changes in the outer layers of the retina, a distorted line of photoreceptors and RPE. (a) The fovea during hospitalization. (b) One of the lesions during hospitalization. (c) The other lesion during hospitalization. (d) The fovea at 5-week follow-up. (e) One of the lesions at 5-week follow-up. (f) The other lesion at 5-week follow-up.

**Figure 3:**
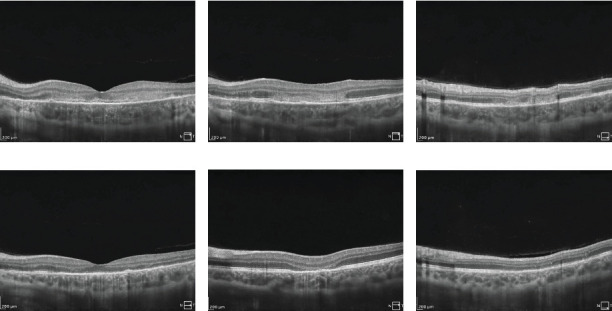
The choroid of the left eye has been cropped in the OCT images. Images showing hyperreflective changes in the outer layers of the retina, a distorted line of photoreceptors and RPE. (a) The fovea with central macular involvement during hospitalization. (b) One of the lesions during hospitalization. (c) The other lesion during hospitalization. (d) The fovea at 5-week follow-up. (e) One of the lesions at 5-week follow-up. (f) The other lesion at 5-week follow-up.

**Figure 4:**
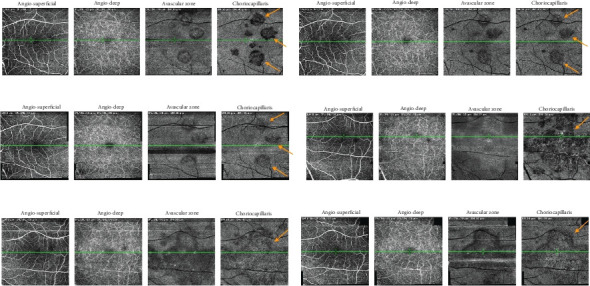
OCTA scans showing plenty of choriocapillaris flow deficits. (a) The right eye during hospitalization. (b) The right eye at 5-week follow-up. (c) The right eye at 14-week follow-up. (d) The left eye during hospitalization. (e) The left eye at 5-week follow-up. (f) The left eye at 14-week follow-up.

**Figure 5:**
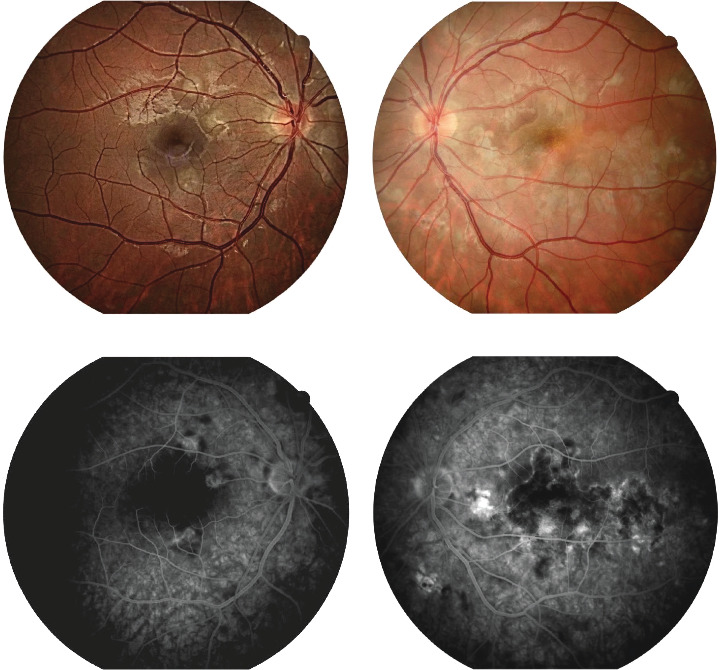
Fluorescein angiography images showing (a) color fundus photography of the right eye with scattered yellow–white lesions. (b) Color fundus photography of the left eye with more numerous, plaque-like lesions forming a diffuse area in the macula. (c) Late phase FA in the right eye with patchy hyperfluorescence and pigment staining. (d) Late phase FA in the left eye with progressive leakage, dye staining, uneven saturation of lesions, and patchy hyperfluorescence.

**Figure 6:**
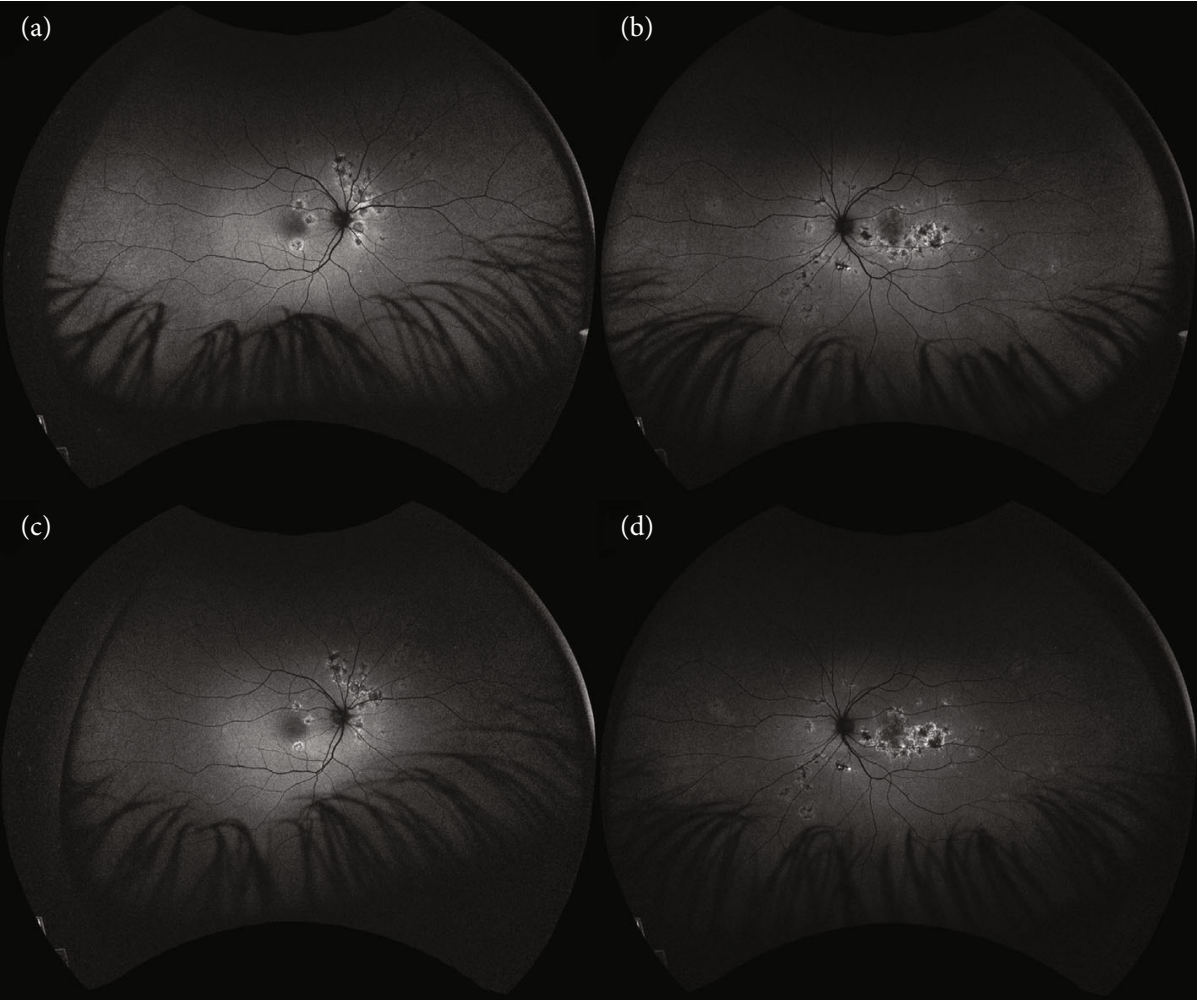
Fundus autofluorescence images showing the right eye (a) and the left eye (b) with hypoautofluorescent lesions with hyperautofluorescence at the periphery of the lesions, during hospitalization. The right eye (c) and the left eye (d) with still present hypoautofluorescent lesions with hyperautofluorescence at the periphery at 5-week follow-up.

**Figure 7:**
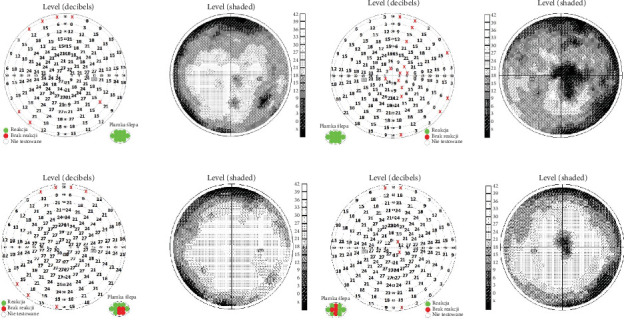
The visual field test of both eyes showing (a) VF of the right eye taken during hospitalization. (b) VF of the left eye taken during hospitalization. (c) VF of the right eye taken at 5-week follow-up. (d) VF of the left eye taken at 5-week follow-up.

**Figure 8:**
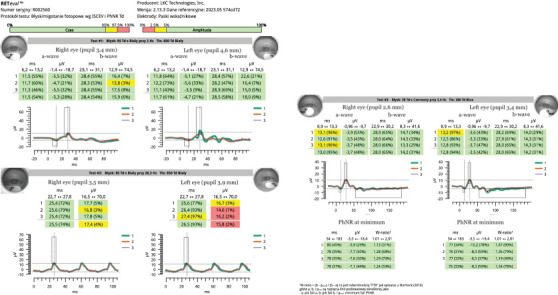
ERG of both eyes at the beginning of the hospitalization. The results of the white flash stimulation at 28.3 Hz included a decrease in wave amplitude to 17.4 *μ*V in the right eye and to 15.8 *μ*V in the left eye and a wave latency of 25.5 ms in the right eye and 26.5 ms in the left eye.

On the day of admission to our department, the patient was already presenting with the chorioretinal phase of the disease. The BCVA in the right eye was 0.9, and in the left eye, it was 0.025. In a physical examination, the anterior segment and vitreous body of both eyes showed no signs of inflammation. In the right eye, scattered yellow–white lesions of the retina and choroid were observed near the optic disc, in the upper hemisphere of the retina, and three foci were present in the macula. In the left eye, there were more numerous, plaque-like lesions, forming a diffuse area of damage, involving the fovea. There were many smaller, scattered lesions in the midperiphery of the retina in the left eye (Figure 1).  Table 1 shows the results of various imaging studies performed on an admission day: optical coherence tomography (OCT) (Figures 2 and 3), OCTA (Figure 4), fluorescein angiography (FA) (Figure 5), fundus autofluorescence (FAF) (Figure 6), VF (Figure 7), and electroretinography (ERG) (Figure 8).

We performed numerous tests that allowed us to make an accurate differential diagnosis. A complete blood count, sedimentation rate, and C-reactive protein tests were performed, with results within normal limits. Antigen tests for SARS-CoV-2, RSV, and Influenza Type A and B infection were negative. We obtained negative results for HIV-1 and HIV-2 antibodies, hepatitis B surface antigen, anti-HBe antibodies, and anti-HCV antibodies, as well as the absence of CMV-DNA, anti-Borrelia burgdorferi IgM/IgG antibodies, negative Quantiferon TB Gold Plus, and negative results for VDRL and TPHA. The angiotensin-converting enzyme level was normal; the chest radiograph was physiological.

The immunogenetic test showed the presence of HLA-B15 and HLA-B35 antigens but excluded the presence of HLA-B7 and HLA-DR2 antigens.

Magnetic resonance imaging (MRI) of the brain was performed to exclude the presence of accompanying intracerebral vasculitis. No perivascular inflammation or other pathological features were observed in the MRI examination. A neurological consultation was then performed, during which Vogt–Koyanagi–Harada syndrome was excluded. Due to the characteristic clinical picture and course of the disease, birdshot retinochoroidopathy, serpiginous choroidopathy, and multiple evanescent white dot syndrome were excluded.

On the day of admission, systemic steroid therapy and antiviral treatment were started. Intravenous dexamethasone at a dose 8 mg was used during hospitalization. Intravenous acyclovir at a dose of 5 mg/kg three times per day was used for 5 days. After the first day of treatment, a significant improvement in visual acuity in the left eye was observed. The BCVA was then 0.9 in the right eye and 0.25 in the left eye. The patient was discharged from the hospital with a recommendation to continue using steroids. Oral methylprednisolone was recommended at a dose of 0.8 mg/kg/day with a slow reduction of steroid therapy for a period of 7 weeks. On the day of discharge from the hospital, the patient's BCVA was 1.0 in the right eye and 0.32 in the left eye.

### 2.2. The First Follow-Up

The first follow-up visit took place 9 days after the end of hospitalization. The patient reported subjective improvement in visual acuity and VF. BCVA in the right eye was 0.9 and 0.5 in the left eye. There were no abnormalities in the physical examination of the anterior segment and vitreous body of both eyes. In the eye fundus of the right and left eyes, light yellow, speckled inflammatory lesions with a smaller area were visible compared to the day of discharge from the hospital. Oral steroidal therapy was continued in slowly decreasing doses over a period of 7 weeks.

### 2.3. The 3-Week Follow-Up

Three weeks after hospitalization, the patient's BCVA was 1.0 in the right eye and 0.9 in the left eye.

### 2.4. The 5-Week Follow-Up

**Figure 9:**
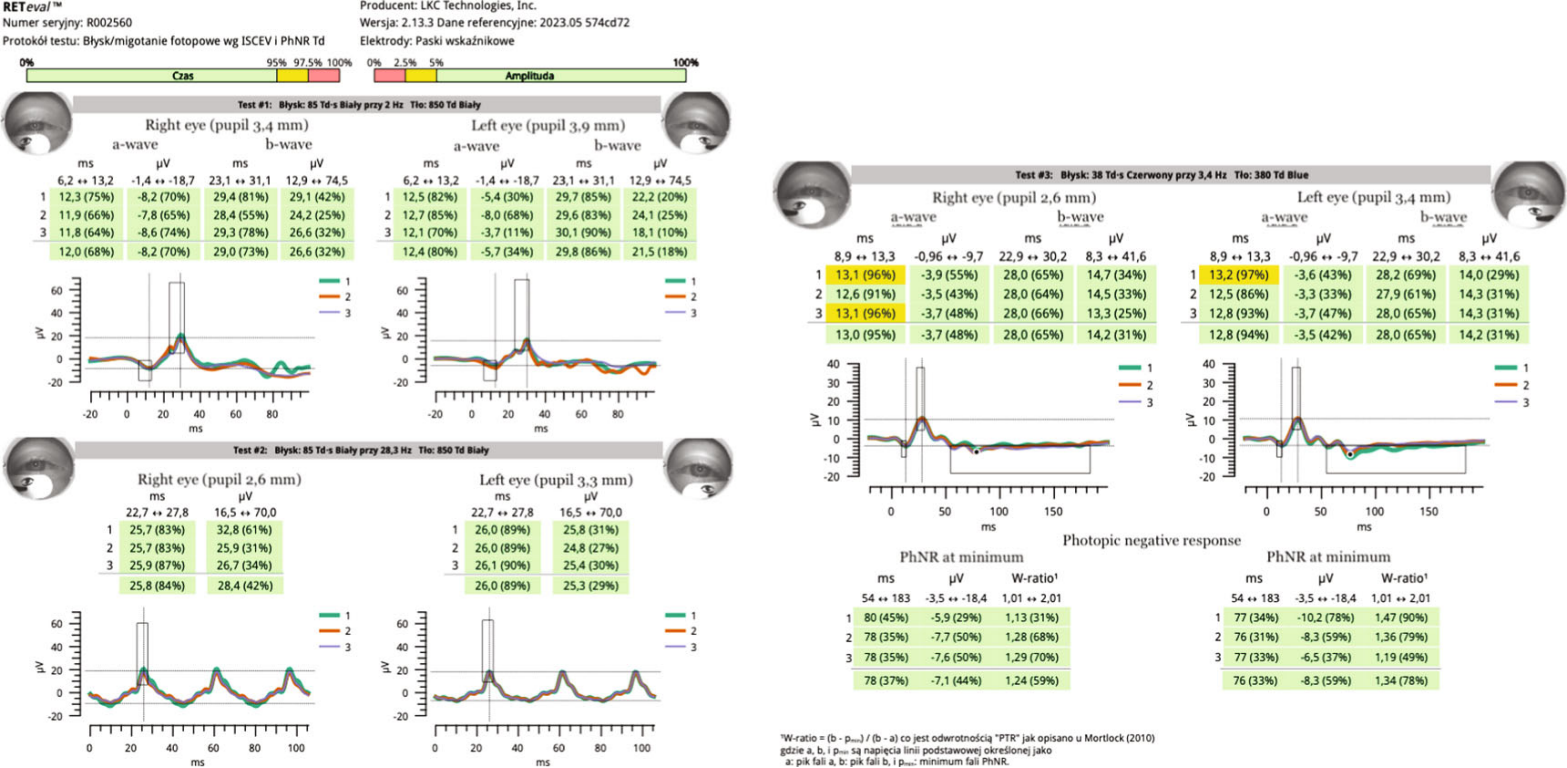
ERG of both eyes taken at 5-week follow-up. The results of the white flash stimulation at 28.3 Hz included an amplitude of 28.4 *μ*V in the right eye and 25.3 *μ*V in the left eye and a wave latency of 25.8 ms in the right eye and 26.0 ms in the left eye.

Five weeks after hospitalization, the patient entered the transitional phase. The patient's visual acuity in both eyes was 1.0. The physical examination of the anterior segment and vitreous body of both eyes remained normal. In the eye fundus of the right and left eyes, light yellow, speckled lesions were visible, smaller in number and in area compared to previous examinations (Figure 1).  Table 2 shows the results of various imaging studies performed during the 5-week follow-up: OCT (Figures 2 and 3), OCTA (Figure 4), FAF (Figure 6), VF (Figure 7), and ERG (Figure 9).

### 2.5. The 14-Week Follow-Up

Fourteen weeks after hospitalization, the patient was still in the transitional phase. BCVA in both eyes was 1.0. OCTA scans reveled a reduction in intensity of choriocapillaris lesions (Figure 4). The disease had not yet reached the resolution phase.

## 3. Discussion

APMPPE is a severe disease. Clinical studies in patients with APMPPE indicate a higher prevalence of tissue antigens HLA-B7 and HLA-DR2 compared to healthy individuals [11, 12]. This suggests an immunogenetic predisposition to the disease. Immunogenetic testing of our patient revealed the presence of HLA-B15 and HLA-B35, while excluding the presence of HLA-B7 and HLA-DR2.

HLA-B15 and HLA-B35 are associated with an increased risk of developing inflammatory diseases. In APMPPE, the presence of this antigen may also indicate a genetic predisposition and a potential link to the body's immune response to infections or other inflammation-triggering factors. We have not found any publications that would discuss the occurrence of these antigens during the APMPPE. Our work may be the beginning of the search for new tissue antigens predisposing to APMPPE. Individuals with HLA-B35 have an increased risk of retinal vasculitis in idiopathic autoimmune choroiditis and kidney inflammation in IgA-associated vasculitis [13, 14]. Increased occurrence of HLA-B15 has been observed in individuals with ankylosing spondylitis [15].

Changes in retinal blood vessels in APMPPE have not been well documented. In our diagnostic workup, we have used multiple imaging techniques for the retinal and choroidal microcirculation. OCT provides detailed information on the structure of the retinal layers, allowing identification of morphological changes such as thickening or damage to the RPE. OCTA focuses on blood vessels, providing insight into the density and integrity of the vessels in the retina and choroid, which is crucial for understanding the pathophysiology of APMPPE. OCTA studies reveal that changes in the density of blood vessels in the macula can suggest ischemia and perfusion disorders that are not always visible using traditional techniques. FA provides information on blood vessel perfusion, but it is less precise in assessing the microcirculation, especially in the context of choroidal capillaries. In contrast to FA, OCTA allows imaging of the microcirculation, which is important for detecting early vascular changes that may precede clinical symptoms.

During hospitalization, in the OCT examination of our patient's retinal microcirculation, the density coefficient of the superficial plexus vessels in the macular area was reduced. After 5 weeks of treatment, this increased by 7.9% in the right eye and 1.2% in the left eye. Similarly, the deep plexus vessel density, which was also decreased during hospitalization, increased by 4.7% in the right eye and 2.6% in the left eye after 5 weeks. These findings indicate a decrease in retinal vessel density during the disease, followed by regeneration and increased density after treatment, confirming the treatment's effectiveness.

To assess the severity of the disease, we performed an ERG to determine the response of retinal photoreceptors. During hospitalization, the ERG showed reduced a- and b-wave amplitudes in both eyes, typically associated with limited loss of retinal function [16]. In the follow-up examination 5 weeks after hospitalization, the a- and b-wave amplitudes normalized in both eyes, indicating an improvement in the electrical response of the retina and resolution of the disease. The ERG was useful in monitoring disease progression and confirming the effectiveness of the treatment.

APMPPE treatment remains controversial, as the disease can be self-limiting. However, in more severe cases, particularly those with systemic involvement or concerning visual loss, pharmacotherapy may be considered to reduce inflammation and mitigate potential complications.

Corticosteroids are used in the management of APMPPE due to their anti-inflammatory properties. The rationale for corticosteroid uses in this condition lies in the potential immune-mediated inflammatory nature of the disease.

Methylprednisolone acts by inhibiting the migration of leukocytes and suppressing cytokine production, which reduces inflammation. It is commonly used in autoimmune or inflammatory conditions affecting the eye, like uveitis, optic neuritis, or central retinal vasculitis, which may overlap with the pathophysiology of APMPPE. Papasavvas et al. [17] proved that the systemic corticosteroid therapy is necessary in a large proportion of cases. Oliveira et al. [8] used methylprednisolone at a dose of 1 mg/kg/day in a patient with macular involvement.

Acyclovir is an antiviral agent, often included in the treatment of APMPPE, particularly if a viral etiology is suspected. While APMPPE is not definitively viral in origin, it has been associated with prior viral infections, suggesting a possible infectious trigger. Acyclovir specifically targets viral replication by inhibiting viral genetic material synthesis. There is no high-level evidence (such as randomized controlled trials) conclusively supporting the use of acyclovir in all cases of APMPPE, but case reports and small series suggest that antivirals may be beneficial in individuals with a known or suspected viral trigger. Brydak-Godowska et al. [18] described the case of 7 patients with APMPPE treated with prednisone, acyclovir, or both, with visual acuity returning in 6 out of 7 people. Chan et al. [19] described the case of an 18-year-old woman with macular involvement treated with intravenous acyclovir and intravitreal ganciclovir.

To summarize, methylprednisolone and acyclovir may be selected for their respective roles in combating severe inflammation and potential viral triggers in APMPPE. While the overall pharmacologic approach remains controversial due to the self-limiting nature of the disease, this combination therapy can be justified in more severe or atypical presentations. Other authors opted not to administer any treatment. Xerii et al. [9] did not use pharmacological treatment for patients with macular involvement, achieving complete patient recovery after 10 months of observation. Similarly, Jakirlic and Harris [4] initially did not treat a patient with postvaccination APMPPE. However, after 42 days of observation, inflammatory changes in the vitreous body developed, and oral prednisone was then successfully administered in tapering doses over a period of 5 weeks [6].

The patient in our case presented with bilateral macular involvement, including the fovea in the left eye. A lower dose of medication, 0.8 mg/kg/day of methylprednisolone, was administered compared to previously used doses in similar cases of APMPPE. Additionally, our patient was treated with intravenous acyclovir, which has been used in only a few reported cases and it is rarely used so far. The patient achieved full visual acuity after 5 weeks of therapy, confirming the effectiveness of the treatment and faster recovery.

APMPPE is typically self-limiting, with most patients recovering vision within weeks to months. However, the long-term prognosis varies based on the severity of the initial episode and any systemic complications. While most recover significantly, some may have permanent scotomas, color vision changes, or slight vision reduction. APMPPE has rare recurrences, particularly in cases with systemic vasculitis or immune issues.

## 4. Conclusion

The presented case of APMPPE emphasizes the crucial role of OCTA in diagnosing the disease and adds novel information by showing, for the first time, changes in retinal vascular density found in OCTA images during the disease course. These changes are a useful tool for treatment, monitoring the disease course, and determining the treatment effectiveness. In severe cases of APMPPE preceded by viral infection, with bilateral macular involvement, using methylprednisolone at a dose of 0.8 mg/kg/day and antiviral treatment has been proven sufficient and effective.

## Figures and Tables

**Table 1:** Results of diagnostic tests (OCT, OCTA, FA, FAF, VF, ERG) in patient with APMPPE performed on an admission day.

**Study type**	**Findings**
OCT	• Active, hyperreflective changes in outer retinal layers • Distorted photoreceptor line and RPE, with choroidal thickening suggesting inflammation (Figures 2 and 3)

OCTA	• Plenty of choriocapillaris flow deficits • Right eye: lesions of choriocapillaris deficits; left eye: diffuse area of choriocapillaris deficits (Figure 4) • Superficial capillary plexus vessel density: right eye 34.3%, left eye 37.1% • Deep capillary plexus vessel density: right eye 38.8%, left eye 38%

FA	• Early hypofluorescence due to decreased choriocapillaris perfusion and RPE thickening • Patchy hyperfluorescence indicating uneven dye saturation of lesions (Figure 5)

FAF	• Hypoautofluorescence at RPE damage sites with hyperautofluorescence at lesion peripheries (Figure 6)

Visual field (VF)	• Right eye: paracentral and confluent arcuate scotomas, narrowing of VF • Left eye: significant generalized decrease in retinal sensitivity, large central scotoma, confluent arcuate scotomas, narrowing of VF lunette like (Figure 7)

Electroretinogram (ERG)	• White flash stimulation (28.3 Hz) showed decreased wave amplitude: right eye 17.4 *μ*V, left eye 15.8 *μ*V • Wave latency: right eye 25.5 ms, left eye 26.5 ms (Figure 8)

**Table 2:** Results of diagnostic tests (OCT, OCTA, FAF, VF, ERG) in patient with APMPPE performed on the 5-week follow-up.

**Study type**	**Findings**
OCT (SOCT REVO NX 130 Optopol, Poland)	• Inflammatory lesions visible but with smaller diameter and thickness compared to hospitalization (Figures 2 and 3)
OCTA	• Reduction in size and intensity of choriocapillaris lesions in the macula • Superficial capillary plexus vessel density: right eye 42.2%, left eye 38.8% • Deep capillary plexus vessel density: right eye 43.5%, left eye 40.6% (Figure 4)
FAF	• Areas of damage to the retinal pigment epithelium with hyperautofluorescence at the periphery of lesions (Figure 6)
VF	• Right eye: confluent arcuate defects • Left eye: increased retinal sensitivity, smaller central scotoma, and arcuate defects • Significant improvement in the patient's field of vision (Figure 7)
ERG	• White flash stimulation (28.3 Hz) showed amplitude: right eye 28.4 *μ*V, left eye 25.3 *μ*V • Wave latency: right eye 25.8 ms, left eye 26.0 ms (Figure 9)

## Data Availability

All data generated or analyzed during this study are included in this published article.

## Nomenclature

APMPPE: acute posterior multifocal placoid pigment epitheliopathy
OCT: optical coherence tomography
OCTA: optical coherence tomography angiography
BCVA: best corrected visual acuity
ERG: electroretinogram
RPE: retinal pigment epithelium
FAF: fundus autofluorescence
CRP: C-reactive protein
SARS-CoV-2: severe acute respiratory syndrome coronavirus 2
RSV: respiratory syncytial virus
HLA: human leukocyte antigens
MRI: magnetic resonance imaging
FA: fluorescein angiography
HIV1: human immunodeficiency virus type 1
HIV2: human immunodeficiency virus type 2
HBV: hepatitis B virus
HCV: hepatitis C virus
